# Supplementary material for: Association between oral microbiome and seven types of cancers in East Asian population: a two-sample Mendelian randomization analysis
Source: Front Mol Biosci. 2023 Nov 21;10:1327893. doi: 10.3389/fmolb.2023.1327893 (PMC10702768; doi:10.3389/fmolb.2023.1327893)
Supplement: Supplementary file 1 [file DataSheet1.ZIP › Supplementary Materials/MR plots of saliva/saliva═╝/Pancreatic cancer/pheno.1040_to_pancreatic cancer_forest.pdf]

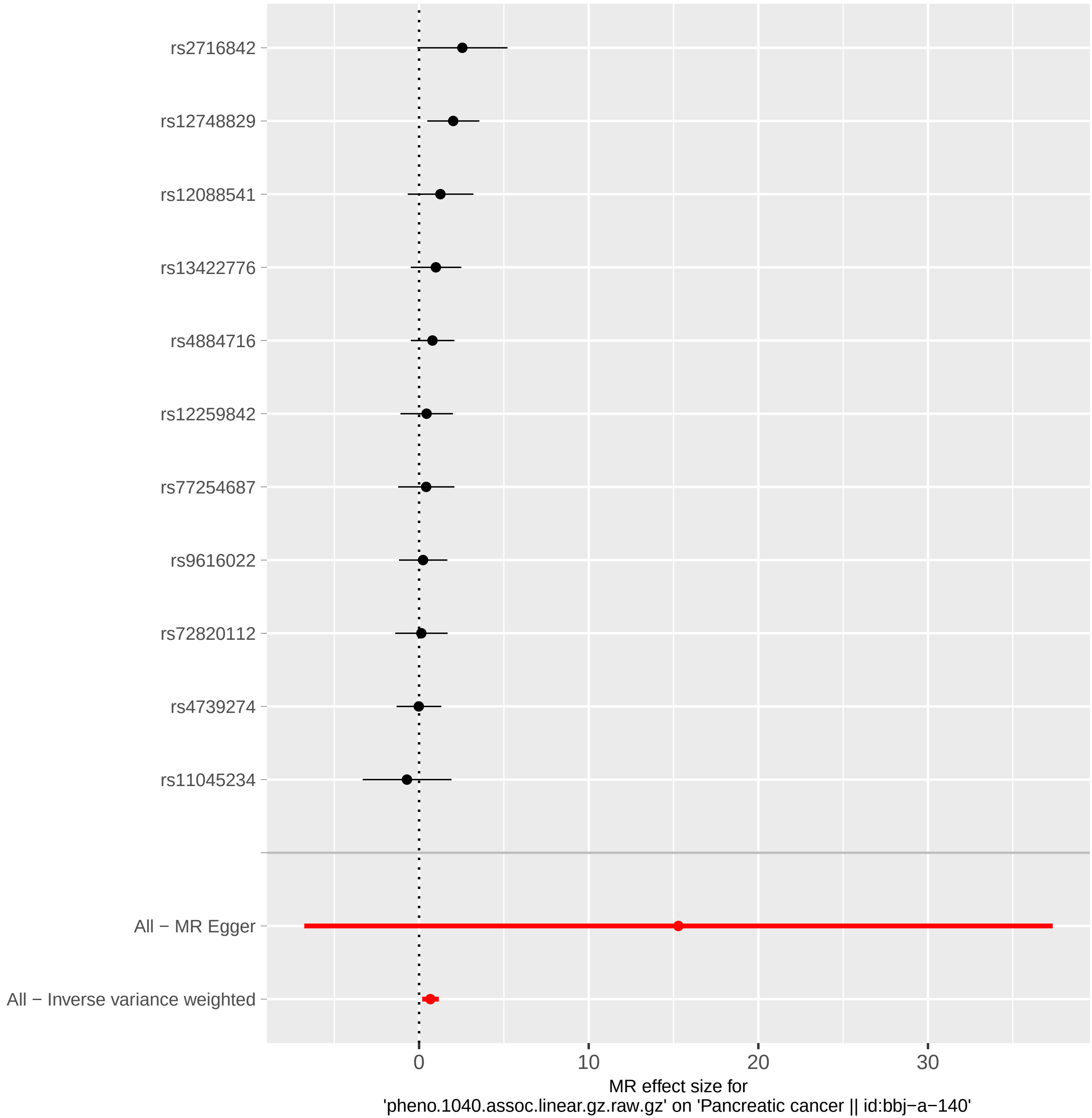

rs2716842
rs12748829
rs12088541
rs13422776
rs4884716
rs12259842
rs77254687
rs9616022
rs72820112
rs4739274
rs11045234
All − MR Egger
All − Inverse variance weighted
0
10
20
30
MR effect size for
'pheno.1040.assoc.linear.gz.raw.gz' on 'Pancreatic cancer || id:bbj−a−140'
